# Supplementary material for: Zika virus persistence in the male macaque reproductive tract
Source: PLoS Negl Trop Dis. 2022 Jul 5;16(7):e0010566. doi: 10.1371/journal.pntd.0010566 (PMC9299295; doi:10.1371/journal.pntd.0010566)
Supplement: S1 Table — All statistical tests performed using R-studio. Only significant and borderline P-values reported here. P value ≤ 0.05 is significant, P value 0.05–0.08 is borderline. CI is confidence interval; Ab is antibody; DPI is days post-inoculation; AUC is area under the curve. 1Intravenous (IV) as reference. (DOCX) [file pntd.0010566.s001.docx]

**S1 Table**

| Dependent Variable | Experimental Condition | Test | Estimate  (95%CI) | P-value |
| --- | --- | --- | --- | --- |
| Viremia positive/negative | Administration of anti-ZIKV Ab | Fisher’s exact | 0  (0.0, 1.19) | 0.03 |
| Peak viremia magnitude | Administration of anti-ZIKV Ab | t-test | 2.03  (0.93, 3.07) | 0.02 |
| Peak viremia (DPI) | Administration of anti-ZIKV Ab | t-test | -9.23  (-20.90, -11.49) | 0.0005 |
| Overall viremia duration | Administration of anti-ZIKV Ab | t-test | -7.69  (-15.86, -8.35) | 0.0001 |
| Peak viremia magnitude | ZIKV Dose | Kruskal Wallis | 19.73 (chi square) | 0.03 |
| Peak viremia magnitude | Route of inoculation^1^ | t-test | 2.62  (0.21, 1.89) | 0.02 |
| Viremia AUC | Reinoculation | t-test | 3.89  (10.68, 43.94) | 0.002 |
| Detection of ZIKV RNA in the male reproductive tract | Route of inoculation^1^ | Fisher’s exact | 8.71  (1.52, 94.17) | 0.01 |
| Detection of ZIKV RNA in epididymis | Route of inoculation^1^ | Fisher’s exact | 13.5  (2.23, 115.66) | 0.001 |
| Detection of ZIKV RNA in epididymis | Administration of anti-ZIKV Ab | Fisher’s exact | 0.073  (0.001, 0.73) | 0.01 |
| Detection of ZIKV RNA in seminal vesicle | Route of inoculation^1^ | Fisher’s exact | 6.11  (1.41, 31.35) | 0.01 |
| Histology score, epididymis | Route of inoculation^1^ | Simple logistic regression | 1.03  (0.36, 1.70) | 0.004 |
| Histology score, prostate gland | Route of inoculation^1^ | Simple logistic regression | 0.56  (0.01, 1.10) | 0.05 |
| Histology score, epididymis | Reinoculation | Simple logistic regression | 1.62  (0.88, 2.36) | 0.002 |
| Histology score, prostate gland | Reinoculation | Simple logistic regression | 0.95  (0.30, 1.61) | 0.01 |
| Histology score, prostate gland | Administration of anti-ZIKV Ab | Simple logistic regression | -1.32  (-1.96, -0.68) | <0.0001 |
