# Supplementary material for: Zika virus persistence in the male macaque reproductive tract
Source: PLoS Negl Trop Dis. 2022 Jul 5;16(7):e0010566. doi: 10.1371/journal.pntd.0010566 (PMC9299295; doi:10.1371/journal.pntd.0010566)
Supplement: S2 Table — Statistical tests performed using R-studio or Graphpad Prism. Only significant P-values reported here. P value ≤ 0.05 is significant, P value of 0.05–0.08 is borderline; CI is confidence interval; DPI is days post-inoculation. (DOCX) [file pntd.0010566.s002.docx]

**S2 Table**

| Dependent variable | Independent variable | Test | ESTIMATE  (95% CI) | P-value |
| --- | --- | --- | --- | --- |
| Detection of ZIKV RNA in epididymis | Magnitude peak viremia | Ordered logistic regression model | 0.24  (0.11, 0.37) | 0.0007 |
| Detection of ZIKV RNA in male reproductive tract | Sexual maturity | Ordered logistic regression model | 0.33  (0.06, 0.61) | 0.02 |
| Detection of ZIKV RNA in epididymis | Sexual maturity | Ordered logistic regression model | 0.71  (0.46, 0.95) | <0.0001 |
| Detection of ZIKV RNA in seminal vesicle | Sexual maturity | Ordered logistic regression model | 0.59  (0.28, 0.88) | 0.0005 |
| Detection of ZIKV RNA in seminal vesicle | Days post-inoculation | Ordered logistic regression model | -0.02  (-0.02, -0.006) | 0.005 |
| Detection of ZIKV RNA in seminal vesicle from 1-20 DPI | Detection of ZIKV RNA in seminal vesicle from 21-40 DPI | Mann-Whitney | - | 0.02 |
| Detection of ZIKV RNA in seminal vesicle from 1-20 DPI | Detection of ZIKV RNA in seminal vesicle from 41-60 DPI | Mann-Whitney | - | 0.03 |
| Histology score epididymis (ZIKV-inoculated) | Histology score epididymis (uninfected controls) | Mann-Whitney | - | 0.02 |
| Histology score prostate (ZIKV-inoculated) | Histology score prostate (uninfected controls) | Mann-Whitney | - | <0.0001 |
| Histology score epididymis | Sexual maturity | Linear model | 0.92  (0.15, 1.68) | 0.02 |
| Histology score prostate | Sexual maturity | Linear model | 1.17  (0.62, 1.71) | 0.0001 |
| Histology score epididymis | Detection of ZIKV RNA in epididymis | Linear model | 1.09  (0.34, 1.84) | 0.01 |
| Histology score epididymis | Days post-inoculation | Linear model | -0.03  (-0.05, -0.007) | 0.02 |
| Histology score prostate | Days post-inoculation | Linear model | -0.02  (-0.05, -0.003) | 0.03 |
