# Supplementary material for: Zika virus persistence in the male macaque reproductive tract
Source: PLoS Negl Trop Dis. 2022 Jul 5;16(7):e0010566. doi: 10.1371/journal.pntd.0010566 (PMC9299295; doi:10.1371/journal.pntd.0010566)
Supplement: S3 Table — (DOCX) [file pntd.0010566.s003.docx]

**S3 Table**:

|  | **Testis** | **Epididymis** | **Seminal vesicle** | **Prostate gland** |
| --- | --- | --- | --- | --- |
| **0** (none) | No significant lesions | No significant lesions | No significant lesions | No significant lesions |
| **1** (minimal) | Lesions minimal and significance questionable (affecting <5% of the visible surface area): Rare perivascular/ peritubular mononuclear infiltrates (less than 3 small foci); and/or mild evidence of sperm stasis (rete testes or efferent ducts exhibit sperm aggregation with debris, macrophages, and multinucleated giant cells +/- engulfed sperm) | Lesions minimal and significance questionable (affecting <5% of the visible surface area): Rare perivascular/ periductular mononuclear infiltrates (less than 3 small foci); and/or mild evidence of sperm stasis (dilated epididymal ducts lacking sperm with replacement by debris, macrophages, and multinucleated giant cells +/- engulfed sperm) | Lesions minimal and significance questionable: Rare perivascular/ peritubular mononuclear infiltrates (less than 3 small foci) | Lesions minimal and significance questionable (affecting <5% of the visible surface area): Rare perivascular/periglandular mononuclear infiltrates (less than 3 small foci); and/or foci of increased fibrous connective tissue |
| **2** (mild) | Mild lesions not observed in control animals (affecting 5-10% of the visible surface area): As above with more frequent perivascular/peritubular mononuclear infiltrates; and/or mild mixed inflammation, hemorrhage/edema; and/or mild evidence of seminiferous tubule degeneration | Mild lesions not observed in control animals (affecting 5-10% of the visible surface area): As above with more frequent perivascular/periductular mononuclear infiltrates; and/or mild mixed inflammation, hemorrhage/edema; and/or mild evidence of ductular epithelial degeneration | Mild lesions not observed in control animals (affecting 5-10% of the visible surface area): As above with more frequent perivascular/peritubular mononuclear infiltrates; and/or mild mixed inflammation, hemorrhage/edema | Mild lesions not observed in control animals (affecting 5-10% of the visible surface area): As above with more frequent perivascular/ periglandular mononuclear infiltrates; and/or mild mixed inflammation, hemorrhage/edema; and/or expansion of glandular lumens by necrotic debris, neutrophils, and mononuclear inflammatory cells; and/or occasional foci of mineralization |
| **3** (moderate) | Moderate lesions not observed in control animals (affecting 10-20% of the visible surface area): As above with multiple larger foci of mixed inflammation; and/or rare, small granulomas; and/or scattered foci of mineralization; and/or seminiferous tubule necrosis | Moderate lesions not observed in control animals (affecting 10-20% of the visible surface area): As above with multiple larger foci of mixed inflammation; and/or rare, small granulomas; and/or scattered foci of mineralization; and/or ductular epithelial necrosis | Moderate lesions not observed in control animals (affecting 10-20% of the visible surface area): As above with multiple larger foci of mixed inflammation; and/or rare, small granulomas; and/or glandular epithelial necrosis | Moderate lesions not observed in control animals (affecting 10-20% of the visible surface area): As above with multiple larger foci of mixed inflammation; and/or rare, small granulomas; and/or scattered foci of mineralization; and/or ductular epithelial necrosis |
| **4** (severe) | Widespread moderate to severe mixed inflammation, necrosis, mineralization and/or granulomas (affecting greater than 20% of the visible surface area) | Widespread moderate to severe mixed inflammation, necrosis, ductular rupture, mineralization and/or granulomas (affecting greater than 20% of the visible surface area) | Widespread moderate to severe mixed inflammation, necrosis, mineralization and/or granulomas (affecting greater than 20% of the visible surface area) | Widespread moderate to severe mixed inflammation, necrosis, glandular rupture, mineralization and/or granulomas (affecting greater than 20% of the visible surface area) |
| **5** (severe +) | Same as "4" but with evidence of chronicity such as frequent, extensive mineralization, replacement of necrotic testicular architecture with fibrous connective tissue, or large, well-organized granulomas or abscesses | Same as "4" but with evidence of chronicity such as frequent, extensive mineralization, replacement of necrotic epididymal architecture with fibrous connective tissue, or large, well-organized granulomas or abscesses | Same as "4" but with evidence of chronicity such as replacement of necrotic seminal vesicular architecture with fibrous connective tissue, or large, well-organized granulomas or abscesses | Same as "4" but with evidence of chronicity such as replacement of necrotic prostatic architecture with fibrous connective tissue, or large, well-organized granulomas or abscesses |
